# A rarity in breast pathology: First recurrent male case of Rosai-Dorfman disease

**DOI:** 10.1016/j.ijscr.2018.10.003

**Published:** 2018-10-08

**Authors:** BenFauzi El-Attrache, Bradley Gluck, Alan Heimann, Edna Kapenhas

**Affiliations:** a Department of Surgery at Stony Brook Southampton Hospital, United States; b Department of Diagnostic Radiology at Stony Brook Southampton Hospital, United States; c Department of Pathology at Stony Brook University Hospital, United States

**Keywords:** Rosai-Dorfman disease, Sinus histiocytosis with massive lymphadenopathy, Rare breast pathology

## Abstract

**Introduction:**

Rosai-Dorfman Disease (RDD) is a rare pathological finding in the breast. Although it is usually palpated as a breast mass, it can be identified on ultrasound or mammogram. The hallmark of the disease is histiocytes with emperipolesis. The case presented in this study represents the first known documented male breast recurrence of RDD and the first association of the disease with pseudoangiomatous stromal hyperplasia (PASH).

**Presentation of case:**

A 55-year-old male, with a prior history of right breast excisional biopsy of a mass in 2015 that showed RDD, presented to our breast clinic for follow up. A breast sonogram showed a hypoechoic mass. Biopsy and excision were performed revealing recurrent RDD along with PASH.

**Discussion:**

RDD is a benign condition that normally presents in lymph nodes but can present in extranodal sites, such as the breast. Location and symptoms guide treatment which can include medical or surgical therapy. PASH of the breast is a benign process that may be amenable to treatment by observation, medical management, or surgical excision. Treatment continues to be controversial given the lack of long term sequelae.

**Conclusion:**

Definitive treatment for RDD of the breast and PASH has not been established. Based on the available data in the literature, our conclusion is that recurrent RDD of the breast can be observed without need for surgical therapy.

## Highlights

• Rosai-Dorfman Disease of the breast is a benign disease with minimal cases.
• Pseudoangiomatous stromal hyperplasia is a benign pathological finding that can be found in men with gynecomastia.
• Asymptomatic recurrent Rosai-Dorfman Disease conservatively managed by observation.
• First recurrent male case of Rosai-Dorfman Disease.
• First case of Rosai-Dorfman Disease associated with pseudoangiomatous stromal hyperplasia.

## 1 Introduction

Rosai-Dorfman Disease (RDD) is a lipid storage disorder that typically presents with painless cervical adenopathy. Extranodal disease to the breast is rare with 20–25 reported cases. Of those, only four are identified in the male breast [1]. This study is a follow up on “A Rarity in Breast Pathology: A Male Case of Rosai-Dorfman Disease and Literature Review” that was published in May 2017 in the International Journal of Surgery Case Reports. The patient presented for follow up two years later, and on clinical exam was noted to have a slightly firm palpable area in the upper outer peri-areolar region of the right breast. After biopsy and excision of the area, RDD was again confirmed. This work has been reported in line with the Surgical Case Report Guidelines (SCARE) criteria [2].

## 2 Presentation of case

A 55 year-old male presents to our institute in April 2017 for follow up. He has a prior history of an excised subareolar right breast lump in March 2015 that showed atypical lymphoid tissue consistent with RDD of the breast. Physical examination revealed a right breast inferior peri-areolar scar with a slightly firm palpable area in the upper outer peri-areolar region. The left breast exam was unremarkable.

**Fig. 1:**
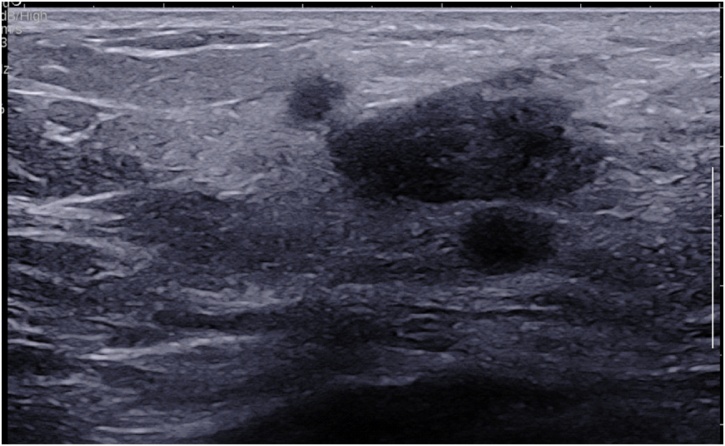
Corresponding to the palpable abnormality in the periareolar upper-outer quadrant of the right breast is an approximately 2.5 × 0.7 cm, irregular, non-circumscribed, hypoechoic mass. Intimately adjacent to this mass are two similar appearing subcentimeter-sized masses.

**Fig. 2:**
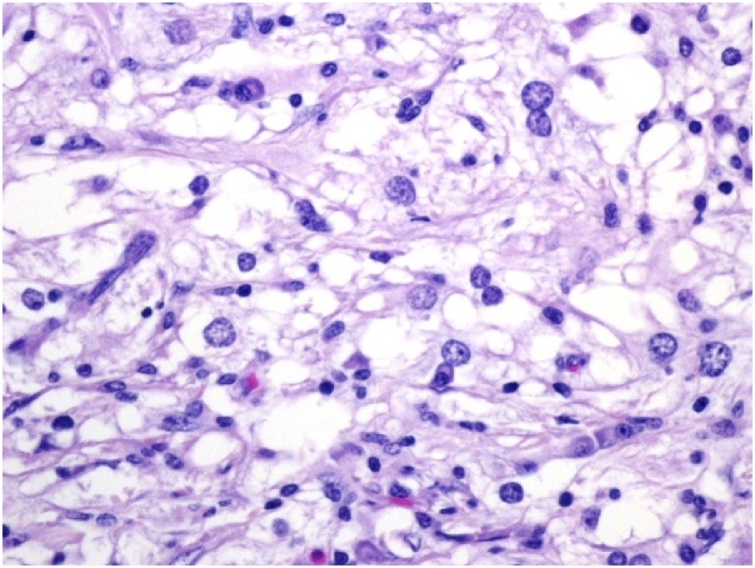
Histiocytes, scattered lymphocytes and plasma cells typical of Rosai-Dorfman Disease (200×).

**Fig. 3:**
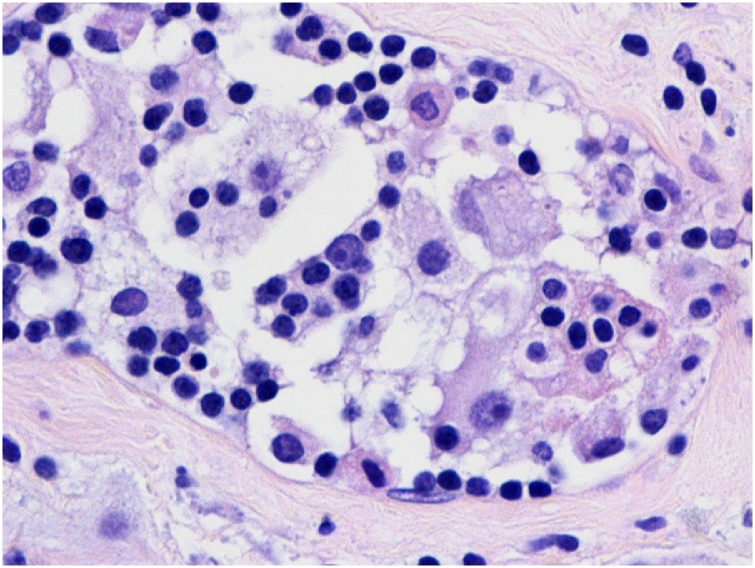
Histiocytes demonstrating emperipolesis with pale nucleus, prominent nucleolus and intracytoplasmic lymphocytes typical of Rosai-Dorfman (600×).

**Fig. 4:**
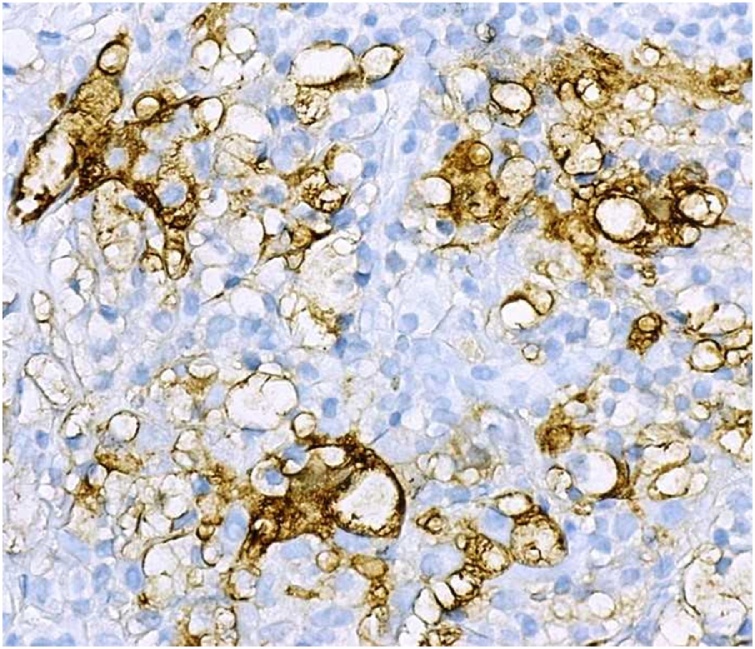
Immunohistochemistry demonstrating positive S100 protein staining (200×).

Diagnostic sonogram revealed an ill-defined 2.5 × 0.7 cm hypoechoic mass with two adjacent nodules (Fig. 1). This was different than the prior ultrasound in 2015 which showed a hyperechoic/isoechoic abnormal echo-texture. A biopsy had shown mixed histiocytic and lymphoplasmacytic infiltrate associated with pseudoangiomatous stromal hyperplasia (PASH). The patient underwent another excision of this area in the right breast and the pathology confirmed the biopsy findings, which were consistent with the patient’s history of RDD (Fig. 2, Fig. 3, Fig. 4).

## 3 Discussion

Defined by Rosai and Dorfman in 1969, RDD is a benign condition that presents in lymph nodes or extranodal sites [3]. Extranodal sites can include: skin, central nervous system (CNS), breast, respiratory tract, and the eye. While extranodal disease is identified in up to 40% of cases, disease confined to the breast is rare [4]. Diagnosis is dependent on the histopathological finding of emperipolesis, which is where intact lymphocytes and immune cells are engulfed by histiocytes [3].

Treatment is based on symptoms and location. In head and neck cases, surgery is done to prevent airway compromise. In the CNS, neurological symptoms can be relieved with excision. Cutaneous RDD can be observed since it is usually asymptomatic. Tumor Necrosis Factor (TNF) inhibitors, corticosteroids, radiation, and chemotherapy have been used with variable success [4].

Although PASH is a benign disease typically found in women, it can present in males with gynecomastia [5]. PASH is identified histologically by stromal proliferation with pseudovascular spaces lined with myofibroblasts [6]. PASH does not have malignant potential; however, it can be identified incidentally in cases of adenocarcinoma [5]. Insufficient excision can lead to recurrence rates of 13–26% [6]. With PASH being an incidental finding in breast adenocarcinoma and with the high recurrence rates, wide local excision may be one of the mainstays of treatment [5]. However, there have been many cases where PASH was managed by observation as an acceptable treatment without any notable long term sequelae. Kareem et al. states that excision may not be necessary if the mass is less than two centimeters or when found on core needle biopsies [7]. These lesions can be subsequently followed with serial imaging and may even be treated medically with tamoxifen [8].

It appears that the size of the lesion and the overall clinical picture needs to be taken into account prior to considering excision, and its management is therefore controversial. To our knowledge, this is the only reported case of recurrent RDD in the male breast. In addition, this is the only case of RDD of the breast that has PASH identified in the histology.

## 4 Conclusion

The rare and variable occurrence of RDD of the breast makes the determination of what is the best treatment option difficult. One could argue that since this breast disease has a benign course, excision is not necessary. With a recurrence and the finding of PASH associated with that recurrence, an argument can be made for and also against excision. Definitive treatment plans have not been demonstrated in the literature. Based on the overall data available about RDD and PASH, our conclusion is that if a patient remains asymptomatic, recurrent RDD of the breast can be managed by observation without re-excision.

## Conflict of interest

There are no conflicts of interest.

## Funding sources

There are no study sponsors or sources of funding.

## Ethical approval

Approval was not required by our institution for this manuscript.

## Consent

Written informed consent was obtained from the patient for publication of this case report and accompanying images. A copy of the written consent is available for review by the Editor-in-Chief of this journal on request.

## Author contribution

1. BenFauzi El-Attrache, DO – writing the paper, data collection and interpretation.

2. Bradley Gluck, MD – interpretation of ultrasound imaging.

3. Alan Heimann, MD – diagnosis of disease from pathology specimen.

4. Edna Kapenhas, MD – study concept, case supervision, and editing.

## Registration of research studies

Research Registry.

UIN: researhregistry3470.

## Guarantor

Edna Kapenhas.

## Provenance and peer review

Not commissioned, externally peer-reviewed.
